# Extracellular Vesicles in Trypanosomatids: Host Cell Communication

**DOI:** 10.3389/fcimb.2020.602502

**Published:** 2020-12-14

**Authors:** Ana Claudia Torrecilhas, Rodrigo Pedro Soares, Sergio Schenkman, Christopher Fernández-Prada, Martin Olivier

**Affiliations:** ^1^Departamento de Ciências Farmacêuticas, Federal University of Sao Paulo (UNIFESP), Diadema, Brazil; ^2^Instituto René Rachou, FIOCRUZ—MG, Belo Horizonte, Brazil; ^3^Departamento de Microbiologia, Imunologia e Parasitologia, UNIFESP, São Paulo, Brazil; ^4^Faculty of Veterinary Medicine, Université de Montréal, St-Hyacinthe, QC, Canada; ^5^The Research Institute of the McGill University Health Centre, McGill University, Montréal, QC, Canada

**Keywords:** *Trypanosoma cruzi*, *Leishmania*, inflammation, insect vector, innate immunity, skin pathology, *Trypanosoma brucei*, extracellular vesicles

## Abstract

*Trypanosoma cruzi, Trypanosoma brucei* and *Leishmania* (Trypanosomatidae: Kinetoplastida) are parasitic protozoan causing Chagas disease, African Trypanosomiasis and Leishmaniases worldwide. They are vector borne diseases transmitted by triatomine bugs, Tsetse fly, and sand flies, respectively. Those diseases cause enormous economic losses and morbidity affecting not only rural and poverty areas but are also spreading to urban areas. During the parasite-host interaction, those organisms release extracellular vesicles (EVs) that are crucial for the immunomodulatory events triggered by the parasites. EVs are involved in cell-cell communication and can act as important pro-inflammatory mediators. Therefore, interface between EVs and host immune responses are crucial for the immunopathological events that those diseases exhibit. Additionally, EVs from these organisms have a role in the invertebrate hosts digestive tracts prior to parasite transmission. This review summarizes the available data on how EVs from those medically important trypanosomatids affect their interaction with vertebrate and invertebrate hosts.

## Introduction

### *Trypanosoma cruzi* and Chagas’ Disease

American trypanosomiasis, popularly known as Chagas’ disease (CD), whose etiologic agent is the flagellated protozoan *Trypanosoma cruzi* was first described by Carlos Chagas in 1909. CD is a neglected disease among the 17 tropical diseases. It is estimated that 8 million people are infected with *T. cruzi* in the world, the majority located in Latin America (WHO, 2019). In addition, 100 million people are at risk of infection and 2,000 deaths each year, circumstances that make CD a serious public health problem. In several Latin American countries, CD is controlled in blood banks and by elimination of vector to prevent transmission (Dias et al., 2002; Cavalcanti et al., 2009). Prevention in some countries like Bolivia is deficient (Coura and Vinas, 2010) and prevalence of the infection in Bolivia has been estimated at 6.8% (Luna et al., 2017) and (Bern, 2015). Nevertheless, oral transmission by ingestion of contaminated food, mainly açai fruit and sugar cane remains a source of new cases everywhere and congenital transmission (Brutus et al., 2008; Dias et al., 2008; Nóbrega et al., 2009; Bastos et al., 2010; Shikanai-Yasuda and Carvalho, 2012).

Despite being an endemic disease in Latin America, cases of CD have been reported in non-endemic regions such as North America (5,500 in Canada and 300,000 infected people in the United States), Europe (80,000 cases), Japan (3,000) and Australia (1,500) (Coura and Vinas, 2010; Perez et al., 2015). The occurrence of CD in these non-endemic regions creates a new epidemiological reality, which is due to population mobility, mainly due to migratory activities, as well as the lack of control in blood banks the source of infection in most of these cases (Coura and Vinas, 2010; Cura et al., 2013; Ries et al., 2016).

The invertebrate host is infected by ingesting trypomastigote forms present in the bloodstream of the mammalian host during hematophagy. In the vector gut, the parasite differentiates in epimastigotes, which subsequently multiply by binary division in the posterior intestine. Along the intestinal tract epimastigotes differentiate into metacyclic-trypomastigotes prior to release with urine and feces during a new blood meal. Such released metacyclic-trypomastigotes are capable of infecting the mammalian host through mucosa or injured regions on the skin. Metacyclic-trypomastigotes enter several cell types forming a parasitophorous vacuole and differentiate into amastigotes (de Carvalho and de Souza, 1989). Cytosolic amastigotes multiply by binary division and after seizing the host cell, differentiate into trypomastigotes, which erupts to the extracellular environment and reaches the bloodstream to continue the life cycle.

### *Trypanosoma brucei* and Human African Trypanosomiasis

*Trypanosoma brucei* is the causative agent of Sleeping sickness or Human African Trypanosomiasis (HAT) and Nagana in cattle. It affects millions of people in countries at sub-Saharan Africa (Buscher et al., 2017). There are three subspecies group; *T. brucei brucei* (the agent of Nagana), *T. brucei gambiense* and *T. brucei rhodesiense*, which cause respectively the chronic and acute forms of the disease. These parasites are transmitted by Tsetse flies (*Glossina* spp.). The insect injects parasites into skin during the blood meal. From the bite, parasites enter the lymphatic system and then pass into the bloodstream. The bloodstream forms persist in blood causing anemia, and reach the central nervous system causing neurological disorders including encephalopathy that can lead to death if untreated (Buscher et al., 2017). Differently from *T. cruzi, T. brucei* is an extracellular parasite and divides in the mammalian bloodstream, in the insects’ gut and salivary glands. The bloodstream trypomastigotes surface is covered by a variant surface glycoprotein (VSG) that forms a coat around the parasite that protects it against host immune defenses (Vickerman and Luckins, 1969). There are about 2500 VSG different genes (Cross et al., 2014). Only one of them is expressed at each time and it is replaced allowing the parasite survival upon the establishment of a robust humoral responses (Mugnier et al., 2016; Aresta-Branco et al., 2019). Once the host establish an immune response, another VSG is expressed allowing the parasite to escape leading to chronic infection. In long-term infections, the parasite crosses the blood brain barrier and causes encephalopathy (Schwede and Carrington, 2010; Rudenko, 2011). In addition, parasite-derived molecules including surface metalloproteases (MSPs), phospholipase-C (PLC) and transferrin receptor (TfR) have been shown to play key roles in the interaction (Ponte-Sucre, 2016).

### Leishmania and Leishmaniasis

Leishmaniases are a spectrum of diseases widely distributed in 98 countries in the world where approximately 350 million people are at risk of infection. It is considered be the World Health Organization (WHO) as the 6^th^ tropical disease. Its control involves basically the treatment of patients with Amphotericin B and Sb-based drugs depending on the country. However, the appearance of side-effects, discontinuation and drug resistance have been increasingly reported hindering chemotherapeutic control in several regions of the world (Kaye and Scott, 2011). Leishmaniasis may exhibit three distinct forms with variations: cutaneous leishmaniasis (CL), mucocutaneous leishmaniasis (MCL) and visceral leishmaniasis (VL). VL can be lethal if not treated in 90% of the cases, whereas CL lesions are of benign course but can be worsened by secondary infection. Although not lethal MCL is the most morbid type of infection destroying the tissues surrounding naso-oro-pharyngeal areas leading to disfiguration of the face. Main viscerotropic species include *Leishmania donovani* and *Leishmania infantum*, in the Old and New World, respectively. Dermotropic species are represented by *Leishmania major/Leishmania tropica* (Old World) and *Leishmania braziliensis*, *Leishmania guyanensis*, and *Leishmania amazonensis* (New World) (Herwaldt, 1999).

*Leishmania* parasites are transmitted through the bite of sand fly vector included in two main genera: *Lutzomyia* and *Phlebotomus* in the New and Old World, respectively (Elnaiem et al., 1994; Vexenat et al., 1994). Sand flies are pool feeder insects, inserting their mouth parts in the skin creating a pool where they take a bloodmeal and parasites are released. During their life cycle *Leishmania* parasites undergo several morpho-physiological modifications from an intracellular amastigote form to extracellular promastigotes in the midgut of the insect vector (Assis et al., 2012). In the sand fly midgut, during a process called metacyclogenesis, parasites differentiate into metacyclic promastigotes that are the infective forms injected in the vertebrate host’s skin. In this proinflammatory milieu formed by sand fly-derived factors including saliva, exosomes, promastigote secretory gel (PSG), and bacteria, several cell types including macrophages and neutrophils are attracted to phagocyte the parasites. Inside those cells, metacyclic promastigotes lose their flagellum and become round-shaped forms so called amastigotes. Those divide until cell rupture and infection of the surrounding cells. During a next blood meal, the sand fly ingests macrophage containing amastigotes and the cycle continues (Peters et al., 2008; Atayde et al., 2015; Atayde et al., 2016; Dey et al., 2018).

## Extracellular Vesicles

EVs are particles formed by a lipid bilayer containing proteins and nucleic acids, which are derived and released by many types of cells (Yáñez-Mó et al., 2015; Devhare and Ray, 2018; Théry et al., 2018; Kao and Papoutsakis, 2019). Therefore, EVs can act as mediators in intercellular communication, either in prokaryotes or eukaryotes organisms (Abels and Breakefield, 2016). These particles modulate short- and long-range events, allowing cells to communicate even at long distances. EVs regulate physiological processes, such as blood coagulation, cell differentiation and inflammation, as well as pathological processes caused cancer, neurological, cardiovascular, and infectious diseases (Raposo and Stoorvogel, 2013; Yáñez-Mó et al., 2015; Kao and Papoutsakis, 2019). EVs are present in several biological fluids, such as: bile, feces, cerebrospinal fluid, nasal, synovial, uterine fluid, breast milk, amniotic fluid, saliva, blood, semen, and urine, as reviewed by Yanez-Mó et al. (2015).

According to ‘‘Minimal Information for Studies of Extracellular Vesicles’’ (MISEV2018), proposed by the International Society for Extracellular Vesicles (ISEV), the term ‘‘extracellular vesicles’’ is referred to all sub-populations of EVs, so it is recommended to use it collectively and universally (Théry et al., 2018). Among the various subtypes of extracellular vesicles, the particles can be defined as exosomes, microvesicles and apoptotic bodies, according to their origin, size, and constituents (Kandasamy et al., 2010; Yáñez-Mó, et al., 2015; Théry et al., 2018).

Exosomes are 50–100 nm in diameter released by eukaryotic cells during differentiation, stimulation and stresses (Mathivanan et al., 2010). They are formed in endosomes as multivesicular bodies and are released after exocytosis through fusion with the plasma membrane (Raposo and Stoorvogel, 2013; Hessvik and Llorente, 2018; Skotland et al., 2019). Exosomes released by B lymphocytes, mast cells, immature dendritic cells, platelets and cytotoxic T lymphocytes act in different physiological and pathological conditions (Théry et al., 2002; Kandasamy et al., 2010; Mathivanan et al., 2010; Raposo and Stoorvogel, 2013; Record, 2014; Record et al., 2014). They promote antigenic presentation (Andre et al., 2001; Wolfers et al., 2001; Théry et al., 2002; Chaput, Andre, et al., 2003a; Chaput, Schartz, et al., 2003b; Théry et al., 2009), signaling in cancer by promoting angiogenesis the immune response and remodeling of the surrounding parenchymal tissues to favor tumor progression (Becker et al., 2016). Exosomes are also involved in the formation of the pre-metastatic niche (Mathivanan et al., 2010; Rak, 2010; Araldi et al., 2012; Raposo and Stoorvogel, 2013; Ichikawa, 2015).

Microvesicles range in size from 50 to 1,000 nm in diameter and are generated from the outer buds of the cell’s plasma membrane. The formation of these particles depends on the redistribution of phosphatidylserine in the membrane bilayer and on the reorganization of the cytoskeleton, mainly through the participation of actin-myosin (Akers et al., 2013). Previous treatment activated platelets with cytochalasin D inhibits the formation of microvesicles by reduction of actin polymerization (Crespin et al., 2009; Li et al., 2013). Microvesicles are involved in several functions. For example, they modulate coagulation and fetomaternal communication (Théry et al., 2018). Apoptotic bodies also formed by external buds from the plasma membrane but have a diameter between 50 and 5,000 nm. They are released during the process of cell death, for example during apoptosis.

These different EV populations contain common sets of proteins, both of cytosolic origin, as heat shock proteins (Hsp 70 and 90) and membrane bound, as CD9, CD37, CD53, CD63, CD81, CD82, tetraspanins, and major histocompatibility complex I (MHC class I) and II (MHC class II). Tetraspanins are a family of proteins composed of four transmembrane domains (Théry et al., 2009; Li et al., 2013; Théry et al., 2018) and considered an exosomal marker, since they were first identified in exosomes derived from B lymphocytes. However, further studies have shown that tetraspanins are also found in several subpopulations of EVs (Zöller, 2009).

EVs are relevant for the communication between pathogens and host cells (Marcilla et al., 2014; Campos et al., 2015; Cwiklinski et al., 2015; Kim et al., 2015; Ancarola et al., 2017; Evans-Osses et al., 2017; Soares et al., 2017). Several protozoan parasites are capable of releasing EVs (Torrecilhas et al., 2012; Marcilla et al., 2014) including *Trichomonas vaginalis, Plasmodium* spp.*, T. brucei*, and *T. cruzi* (da Silveira et al., 1979; Gonçalves et al., 1991; Trocoli Torrecilhas et al., 2009; Martin-Jaular et al., 2011; Cestari et al., 2012; Regev-Rudzki et al., 2013; Szempruch et al., 2016a; Szempruch et al., 2016b; Evans-Osses et al., 2017; Ofir-Birin et al., 2017; Ramirez et al., 2017; Ofir-Birin and Regev-Rudzki, 2019; Rossi et al., 2019; Cronemberger-Andrade et al., 2020), *Leishmania* spp. (Olivier et al., 2012; Hassani and Olivier, 2013; Hassani et al., 2014; Atayde et al., 2015; Atayde et al., 2016; Atayde et al., 2019a; Atayde et al., 2019b), and *Toxoplasma gondii* (Silva et al., 2018). These EVs have immunomodulating functions and affects the fate of the parasite in the host body (Torrecilhas et al., 2012; Campos et al., 2015; Borges et al., 2016).

## *T. cruzi* EVs

**Figure 1:**
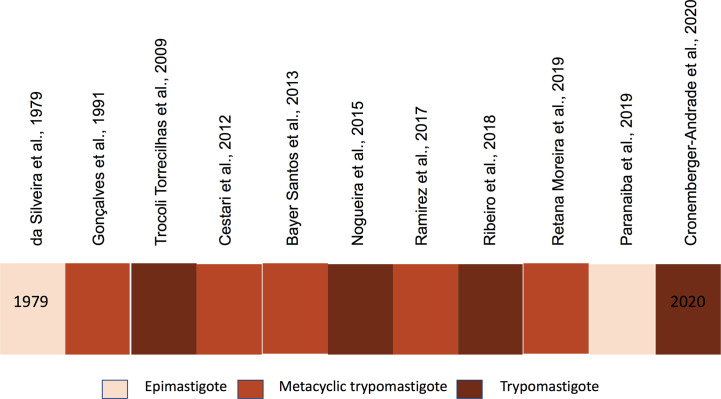
Timeline of *Trypanosoma cruzi* EVs studies.

EVs are produced by the different life stages of *T. cruzi* (da Silveira et al., 1979; Gonçalves et al., 1991; Cestari et al., 2012; Bayer-Santos et al., 2013; Nogueira et al., 2015; Ramirez et al., 2017; Caeiro et al., 2018; Ribeiro et al., 2018; Paranaiba et al., 2019; Rossi et al., 2019) and participate in parasite host interactions (Marcilla et al., 2014; De Pablos et al., 2016; Diaz Lozano et al., 2017; Ramirez et al., 2017; Wyllie and Ramirez, 2017; de Pablos Torro et al., 2018; Retana Moreira et al., 2019; Cronemberger-Andrade et al., 2020). **Figure 1** illustrates EVs in different *T. cruzi* life stages. The lack of studies of amastigote EVs is expected since it’s intracellular location and the secreted material as it is often contaminated with host cell constituents. In both epimastigotes and trypomastigotes, EVs contain several surface components of the parasite involved in the parasite adhesion, invasion, and migration in the vector’s gut (Nogueira et al., 2015; Ribeiro et al., 2018; Paranaiba et al., 2019; Cronemberger-Andrade et al., 2020). Trypomastigote EVs modulates *T. cruzi* infection and affect the protozoan ability to enter and escape the parasitophorous vacuole. Several signaling cascades are activated by EV components and modulate the cell responses (Burleigh and Andrews, 1995a; Burleigh and Andrews, 1995b; Burleigh et al., 1997; Burleigh and Andrews, 1998; Caler et al., 1998; Morty et al., 1999; Kima et al., 2000). For example, EVs affects host cell actin filaments, which allows migration of lysosomes and formation of the parasitophorous vacuole required for parasite internalization (Schenkman et al., 1992; Schenkman and Eichinger, 1993; Schenkman et al., 1993; Schenkman et al., 1994; Fernandes et al., 2011a; Fernandes et al., 2011b; Barrias et al., 2019; Reignault et al., 2019). EVs also modulate the invasion of metacyclic-trypomastigotes by inducing host cell signaling through tyrosine phosphorylation and nucleation of actin filaments (Yoshida and Cortez, 2008).

### Proteomic Analysis of *T. cruzi* EVs

EVs isolated from *T. cruzi* trypomastigotes derived from mammalian cells contain most of the parasite cell-surface proteins, including major surface glycoproteins that resemble to mucins (Ribeiro et al., 2018) (**Table 1**). These glycoproteins are encoded by more than a thousand genes many of them containing heterogenous sequences rich in proline and threonine with N-acetyl glucosamine *O*-linked oligosaccharides that can be either sialylated by the parasite trans-sialidase (TS), or modified by α-galactose (McConville et al., 1990; Almeida et al., 2000; Soares et al., 2012). These carbohydrate moieties are involved in the interaction of the parasite with mammalian host cells (Buscaglia et al., 1998; Almeida et al., 2000; Almeida and Gazzinelli, 2001; Buscaglia et al., 2004; Lantos et al., 2016).

**Table 1:** Extracellular vesicles isolated from Trypanosomatid: features, cargoes, effects on host parasite interaction.

Trypanosomatid	Cargoes/molecules	Effect on host	References
*T. cruzi*	Trans-sialidase Mucin Cruzipain GP85 RNA* GP82** Lipids*** SAPA MASP	i) Induce innate immune response *via* TLR2. ii) Increase Invasion of the parasite iii) Parasites escaping the complement attack modulate TGF-β-bearing EVs released from host cells. iv) Immunomodulation, cell attachment and regulation of host complement system.	(Cestari et al., 2012; Campos et al., 2015; Nogueira et al., 2015; Ramirez et al., 2017; Ribeiro et al., 2018)
*T. brucei*	VSG Protease MSPs PLC	i) Evasion from the host immune system and increase invasion BBB.	(Szempruch et al., 2016)
*L. donovani* *L. infantum* *L. mexicana*	GP63 LPG HSP	i) Parasite evasion by altering complement mediated lysis and promoting parasite phagocytosis.	(Silverman et al. 2010a; Hassani et al., 2011)

MSPs, Major surface proteases; PLC, phospholipase-C; BBB, Blood Brain Barrier; VSG, Variant Surface glycoprotein; LPG, lipophosphoglycan. *RNA; ** GP82 and ***Lipid, are unpublished data. SAPA, Shed-Acute-phase-antigen; MASP, Mucin-Associated Surface Protein.

Trypomastigote EVs also contain N-linked glycoproteins, which are encoded by hundreds of different genes (TS/GP85 superfamily) (Ribeiro et al., 2018) (**Table 1**). The family encodes mostly proteins known as Tc85, GP85, complement-regulatory protein (CRP), flagellum-associated protein, 85 kDa surface antigen, c71 surface protein (Freitas et al., 2011). It is proposed that their lectin like domains in the C-terminal domain can interact with different types of surface molecules of host cells. For example, some of these glycoproteins interact with cytokeratins exposed in the mammalian surface. The superfamily includes a minor population of proteins with trans-sialidase (TS) activity, some of them with an additional 12 amino acid repeats in the C-terminus named shed-acute-phase-antigen (SAPA). There are two distinct populations of *trans*-sialidase differentiated by the substitution of a tyrosine residue by a histidine in the active site of the sialidase domain. This substitution abrogates the hydrolytic activity, but the protein is still able to bind sialylated residues. One of the main targets of these inactive and active trans-sialidase is the CD43, a sialoglycoprotein in hemopoietic cells (Freire-de-Lima et al., 2010), which particularly relevant in the myocarditis development in experimental CD (Alisson-Silva et al., 2019). Mucins and TS/GP85 glycoproteins are associated to the surface of *T. cruzi* through glycosylphosphatidylinositol (GPI) anchors (McConville et al., 1990; Pontes de Carvalho et al., 1993; Schenkman et al., 1993; Schneider et al., 1993; Izquierdo et al., 2015). The GPI moiety is recognized by Toll 2 (TLR2) and 4 (TLR4) receptors in immune cells (Camargo et al., 1997; Almeida et al., 1999; Almeida et al., 2000; Almeida and Gazzinelli, 2001; Campos et al., 2001; Procópio et al., 2002; Campos et al., 2004; Monteiro et al., 2006). EVs, which carry these GPI-containing molecules are able to induce inflammatory responses in murine macrophages *via* TLR2, triggering the synthesis of pro-inflammatory cytokines, such as TNF-α and IL-12, in addition to the production of nitric oxide (NO) (Nogueira et al., 2015; Cronemberger-Andrade et al., 2020). Consistent with these observations, EVs from Y strain also triggered the production of those cytokines and lipid body formation *via* Prostaglandin E2 (PGE2) (Lovo-Martins et al., 2018). Furthermore, other constituents such as the *T. cruzi* Trypomastigote Alanine Valine and Serine rich proteins (TcTASV-C) might also induce EVs signaling as it is largely present in EVs from trypomastigotes from different DTUs (I, II, and VI) (de Pablos Torro et al., 2018).

Cysteine-proteases such as cruzipain and the surface metalloproteases, named GP63 that modulate several processes in the host cells are also present in trypomastigotes EVs. These proteases are involved in several process affecting the infection, including the process of parasite internalization and survival (Scharfstein and Lima, 2008; Alvarez et al., 2012; Watanabe Costa et al., 2016). In addition, cruzipain is also involved in the activation of the host’s immune system (Duschak and Couto, 2009; Acosta et al., 2012; Soprano et al., 2018). Therefore, these peptidases can act independently of the presence of the parasite on the host through circulating EVs. Proteins from cytoskeletal proteins, such as tubulin, heat shock proteins, and other soluble proteins, were also detected in the total EV fractions (Ribeiro et al., 2018).

Another class of proteins found in some proteomic analysis of EVs are cytoskeleton-related proteins, such α- and ß-tubulin, kinesins, and myosins. These cytoskeleton proteins are usually of exosomes from eukaryotic cells. We also identified other proteins related to exosomes and vacuoles, such as heat-shock protein 85 (HSP85), elongation factor 1-alpha, glyceraldehyde 3-phosphate dehydrogenase (GAPDH), vacuolar ATP synthase subunit B, and tetratric-peptide-repeat (TPR) protein (Théry et al., 2018; Doyle and Wang, 2019).

EVs of metacyclic-trypomastigotes also contain major surface components of the parasite, including the gp35/50 mucin-like glycoproteins and members of trans-sialidase family of glycoproteins (Bayer-Santos et al., 2013). These surface components participate in the cell adhesion and invasion of the metacyclic-trypomastigotes (Schenkman et al., 1993; Santori et al., 1996; Neira et al., 2002; Neira et al., 2003; Eickhoff et al., 2010; Cortez et al., 2012; Cortez et al., 2014; Cordero et al., 2019). Gp82 glycoprotein, a metacyclic specific member of the gp85/trans-sialidase family activates mTOR signaling cascades that allow lysosome migration and fusion with the parasitophorous vacuole producing acidification and parasite escape to the cytosol (Cortez et al., 2012; Cortez et al., 2014; Cordero et al., 2019). It also binds to LAMP-2 receptor favoring parasite internalization (Andrade and Andrews, 2005; Albertti et al., 2010; Andrade, 2019). However, previous incubation with EVs released by metacyclic-trypomastigotes decrease invasion of HeLa cells mediated by gp90, another member of the family (Yoshida et al., 1990) and inhibits cell invasion (Rodrigues et al., 2017). These proteins are also released in the soluble form by the endogenous activity of a GPI phospholipase C activity (Schenkman et al., 1988). Therefore, EVs in this stage provide signals that can either increase up to10 times cell invasion. One possibility is that this combination provides specific responses among different host cells and tissues. EVs from epimastigote and metacyclic-trypomastigote forms also contain mucin-like surface glycoproteins GP35/50, typical of the insect stages of the parasite (Schenkman et al., 1993; Bayer-Santos et al., 2013). These molecules act as adhesion molecule in the interaction of the parasite with the host (Ruiz et al., 1993). Therefore, GP35/50 in EVs could modulate their interaction with host cells. Another protein in the epimastigote EVs is the flagellum calcium binding protein (FCaBP), which is linked to internal membrane leaf of the parasite and modulates several proteins through Ca^2+^ signaling (Buchanan et al., 2005). In fact, microvesicles isolated from metacyclic-trypomastigotes forms enhance communication between *T. cruzi* and host cell promoting invasion (Ramirez et al., 2017). These vesicles inhibit complement-mediated lysis and enhance invasion of Vero cells.

### EVs Cargoes Lipids and Nucleic Acids

Studies of non-protein components in EVs are still in their infancy and a limited number of publications is available for trypanosomatids. This provides a promising field to be explored by researchers. Purified EVs of different *T. cruzi* isolates contains phospholipids including cardiolipin, sphingomyelin, phosphatidylcholine, phosphatidylinositol, phosphatidylcholine, phosphatidylethanolamine and phosphatidylserine. Also, they possess an unidentified non-polar component, as detected after extraction and thin-layer chromatography (unpublished). Several fatty acids were detected after extraction of total lipids by gas chromatography and mass spectroscopy (GC-MS) with a large variation (25 to 8) according to the strain. The four common compounds species were: 5-octadecene, 1-octadecene, 1-hexadecene, and 3-heptadecene with 1-Docosene, But-2-enoic acid, amide, 3-methyl-N-metallyl-, heneicosane, tritetracontane found in the Y strain but not in the YuYu (unpublished). As the fatty acids are critical to induce pro-inflammatory responses, these EV components may be important for TLR activation. We also detected RNA, but not DNA in EVs isolated from trypomastigote but their nature and composition should be further investigated. Epimastigotes were also found to release fragments of tRNA that could modify host cells behavior (Garcia-Silva et al., 2014).

### Internalization of EVs Into the Target Cells

Initially, our group showed that *T. cruzi* EVs could be rapidly internalized *in vitro* by cells suggesting that they could also activate intracellular pathways in the host (Torrecilhas et al., 2012). Further, we showed that those EVs could also act as TLR2 agonists suggesting that perhaps both mechanisms could occur (Nogueira et al., 2015). Recently, *T. cruzi* EVs incubated with Toll-like-receptor 2 (TLR2)-transfected CHO cells increased invasion by the parasite. In parallel, they elicit the translocation of NF-κB and gene expression of proinflammatory cytokines (TNF-α, IL-6 and IL-1β), and STAT-1 and STAT-3 signaling pathways (Cronemberger-Andrade et al., 2020). Since GPI-anchored proteins in EVs are TLR2 ligands, this activation is probably one of the mechanisms triggered by EVs.

### Role of *T. cruzi* EVs on Host Responses

Incubation of *T. cruzi* EVs, or parasite infection of host cells induce several responses, including the increased secretion of EVs by host cells (Ramirez et al., 2017; Choudhuri and Garg, 2020; Cronemberger-Andrade et al., 2020). The host EVs contain some parasite antigens and nucleic acids that possible elicit activation of intracellular Toll receptors and increase the inflammatory response. Interestingly, the presence of the remaining parasite DNA processed by Poly(ADP-ribose) polymerase 1 (PARP1) and cyclic GMP-AMP synthase (cGAS) amplified the inflammatory signal.

The interaction of trypomastigote EVs with host cells with epithelial cells or macrophages occurs rapidly and after 15 min parasite proteins are detected by specific antibodies and increase in cytosolic Ca^2+^ (unpublished). This finding is related to increased invasion by the parasite after incubation with the parasite EVs (Cronemberger-Andrade et al., 2020) in agreement with the requirement for Ca^2+^ mobilization during cell invasion (Burleigh and Andrews, 1998). The EVs could also affect the host cell surface cytoskeleton and that might facilitate parasite internalization.

### *In Vivo* Studies

Mice that received EVs prior to infection developed severe heart pathology, with intense inflammatory reaction and higher number of intracellular amastigotes (Trocoli Torrecilhas et al., 2009). The inflammatory infiltrates contain CD4+ and CD8+ T lymphocytes and macrophages and decrease of iNOS spots expression. Also, IL-4 and IL-10 mRNAs in the heart from animals treated with EVs isolated from parasites. The molecules present on EVs act as primers of the host immune system, facilitating the establishment of the infection and inflammation in the animal model. The modulation of prostaglandin is also modulated by pre-injection of parasite EVs (Lovo-Martins et al., 2018).

*T. cruzi* EVs derived from epimastigotes were evaluated during interaction of the parasite with triatomine bugs *Rhodnius prolixus* and *Triatoma infestans*. EVs were artificially offered to the insects prior to infection with epimastigotes. Pre-feeding with EVs delayed early parasite migration to the rectum in *R. prolixus*, but not in *T. infestans*, affecting parasite-host interaction during the initial events of infection in the invertebrate’s gut (Paranaiba et al., 2019). However, the mechanisms underlying parasite retainment remain to be explored.

Therefore, the release of EVs reflects a strategy developed by the parasite to secrete its main surface components that act, for example, facilitating processes such as adhesion and invasion to the host cell (Fernandes, Andrade, et al., 2011a; Fernandes, Cortez, et al., 2011b; Cronemberger-Andrade et al., 2020), as well as evading the host immune system (Cestari et al., 2012). EVs modify the host allowing a more favorable environment for parasite survival and, consequently, to promote infection and possible disease progression (Trocoli Torrecilhas et al., 2009; Cestari et al., 2012; Torrecilhas et al., 2012). It is relevant that different parasite isolates of *T. cruzi* showed surface variations, and most likely different EVs population, that influence the differential activation of the immune response in the host. In fact, EVs of two different strains of the parasite (Y and YuYu) have qualitative and quantitative differences in content, which directly interfere with virulence and the rate of infectivity in different cell types (Ribeiro et al., 2018).

Our finding that several glycoconjugates and other virulence factors are released as main components of EVs, indicates that these vesicles could play a major role in the interaction with host cells. Those key elements may represent novel targets for chemotherapy and vaccines.

### *Trypanosoma brucei* EVs

*T. brucei* bloodstream forms nanotubes originated from the flagellar pocket that breakdown into EVs that contain molecules that are present in the parasite surface (Geiger et al., 2010). These EVs contain mostly VSGs but are particularly enriched in flagellar proteins and proteins involved in the parasite virulence factors (Szempruch et al., 2016a; Szempruch et al., 2016b). These EVs are in the range of 50 to 100 nm in diameter, which correspond to exosomes size. The proteins identified in the secretome of *T. b. rhodesiensis* are also related to the parasite survival strategies as they contain the serum resistance-associated protein (SRA) that allow the parasite to survive in human blood (Geiger et al., 2010). It has been shown that EVs derived from *T. brucei* are highly fusogenic and transfer lipids and VSG glycoproteins to erythrocytes. This alteration in erythrocytes membrane results in its phagocytosis by macrophages in the liver and spleen leading to anemia (Szempruch et al., 2016a; Szempruch et al., 2016b). The EVs released by the parasite may provide additional cues for the parasite scape from the host. For example, VSGs and parasite DNA containing CpG, present in EVs, are recognized by SRA and Toll-like receptor 9, respectively. These interactions could potentiate a strong immune response and modulate macrophage and dendritic cells to activate TNF-α, IL-6, and IL-12 (Stijlemans et al., 2016). *T. brucei* EVs are also proposed to modulate changes in the vascular endothelium (Varikuti et al., 2020).

Additionally, microvesicles (MVs) isolated from *T. brucei* infected hosts induce a progression meningo-encephalitic late stage (S2), inflammatory processes and modulation in astrocytes that resembled the one produced by IFN-γ, a central mechanism in HAT pathogenesis (Dozio et al., 2019).

*T. brucei* has at least two types of EVs. One, that is continuously released by the parasite, and the other that occurs upon parasite stress, as those affecting RNA transcription and processing. In these parasites, pre-mRNAs are transcribed through long polycistronic gene arrays and further processed by trans-splicing with a spliced leader (SL) exon and by polyadenylation (Clayton, 2019). The spliced leader (SL) RNA is processed in the cytosol and upon trans-splicing inhibition is incorporated into multivesicular bodies through the endosomal sorting complexes required for transport (ESCRT), in a similar way as microRNA is secreted in exosomes of mammalian cells. These exosomes have been shown to affect the motility pattern of the parasite itself (Eliaz et al., 2017). These EVs are internalized by insect form (procyclics of *T. brucei* in the insect host and are key components in the parasite-parasite communication. Taken together, the release of *T. brucei* EVs plays a role in cell-cell communication with the host and among themselves and open new insights to development new potential therapeutic targets or diagnostic markers.

## Biogenesis of EVs in Trypanosomes

Very little is known how the EVs are formed and biogenesis whether they are released in special situations, for example during parasite differentiation. Recently, it has been proposed that the parasite surface resembles a quilt with different types of surface proteins distributed in separate patches (Mucci et al., 2017). The GP85/Trans-sialidase proteins form surface aggregates exclude the mucin-like glycoproteins and each patch might be enriched in different groups of EVs either as a consequence of their secretory pathway or biophysical properties (Niyogi et al., 2014). Therefore, it is expected that different EVs might coexist and affect differently the host cells. Many orthologs of the secretory machinery of eukaryote cells (ESCRT machinery) have been described in *T. brucei* (Silverman et al., 2013) and it was shown that inhibiting Vsp36 (an ESCRT component) compromises EVs secretion, but not of nanotube derived EVs (Eliaz et al., 2017). Therefore, different mechanisms of secretion may occur for these distinct types of particles that are also biochemically distinct and functions. How and whether the release of EVs from the membrane is regulated is still a matter of investigation.

### *Leishmania* EVs

As well covered in the previous sections, parasites of the *Leishmania* genus -being also trypanosomatids are known to secrete proteins *via* their endoplasmic reticulum and Golgi apparatus (McConville et al., 2002; Corrales et al., 2010) and flagellar pocket (Field et al., 2007). In this context, several important virulence factors of *Leishmania* (e.g. Zinc-metalloprotease GP63 and LPG) can be released within the insect vector’s gut and inoculated to the mammalian host during the sand fly’s blood meal (Yao et al., 2003; Joshi et al., 2005). Markedly, as previously discussed for *T. cruzi* and *T. brucei*, *Leishmania* parasites require a non-conventional protein secretion system to release proteins lacking a signal peptide. In this way, exosomes have been found to be a selective and efficient pathway for proteins to leave *Leishmania* (Théry et al., 2009; Atayde et al., 2015). In fact, the great majority of *Leishmania* species studied to date revealed that only 5-9% of exosomal proteins have a signal peptide (Silverman et al., 2008; Atyame Nten et al., 2010; Geiger et al., 2010).

Since 2008, several studies reported the release of exosomes by various species of *Leishmania* when cultured *in vitro*, as well as during their replication and development into metacyclic *Leishmania* promastigotes. Notably, in both cases, *in vitro* or *in vivo* produced *Leishmania* exosomes/EVs strongly contributed to enrich the parasite population with major virulence factors such as GP63. Moreover, exosomes have shown to alter myeloid cell signaling and microbicidal functions as strongly as whole promastigotes (Silverman et al., 2010a; Silverman et al., 2010b; Hassani et al., 2014; Atayde et al., 2015). The growing knowledge on leishmanial exosomes highlights the critical role of these extracellular vesicles not only in the infectious process but also in the progression of the parasites within the mammalian host leading to the various leishmaniasis-related pathologies.

Olivier’s initial observation of GP63 clustered within vesicles being transferred to the macrophage led us to study whether *Leishmania* may produce EVs, thus including exosomes (Gomez et al., 2009; Gomez and Olivier, 2010). Markedly, studying the temperature-induced exoproteome of *Leishmania mexicana*, we obtained our first clear evidence demonstrating that *Leishmania* parasites can release small vesicles (Hassani et al., 2011). However, Reiner’s laboratory was the first one to provide a clear demonstration of *Leishmania* parasites secreting well characterized exosomes (Silverman et al., 2010a).

During Olivier’s lab first study, they found that by emulating the transition of the parasite from vector to the mammalian host, the temperature shift was inducing an increase of proteins secretion *via* exosome-like vesicles on the surface of the parasite. Proteomic analysis revealed that almost all *Leishmania* exosomal proteins lacking a signal peptide were secreted in a non-conventional manner (Hassani et al., 2011). Of utmost interest, it was found that those EVs showed similar capacities as whole promastigotes to hijack host macrophage signaling and functions, in part by inducing host phosphor-tyrosine protein (PTP) negative regulatory mechanisms. Using GP63-deficient *Leishmania major* parasites, we demonstrated that the absence of this virulence factor, which is greatly enriched in wild-type *L. major* exosomes, was almost completely abrogating their capacity to modulate host immune response comparatively to its wild-type counterpart (Hassani et al., 2014). This is different from *L. infantum* LPG1 -/- mutants, where the lack of this glycoconjugate did not affect NO and cytokine induction by its EVs (Nogueira et al., 2020). This further demonstrates the critical role played by *Leishmania* virulence factors enriched in leishmanial exosomes. Previous works reported by Silverman and colleagues were seminal to strengthen the role of *Leishmania* EVs as macrophage immunomodulators (Silverman et al., 2010a; Silverman et al., 2010b) such as for instance, to modify IFNγ-induced cytokines secretion by human monocytes cultured in the presence of *L. donovani* vesicles. Moreover, in an *in vivo* context, CD4^+^ lymphocytes from mice inoculated with leishmanial exosomes were more prone to produce immunosuppressive cytokines (i.e. IL-10, IL-4) and exacerbate pathology (Silverman et al., 2010b). More recently, EVs from New-World, dermotropic *L. amazonensis* were found to be more pro-inflammatory, triggering the production of NO, IL-6 and TNF-α in a TLR4/TLR2/NF-κB-dependent fashion (Nogueira et al., 2020). On the other hand, in the same study, EVs from *L. infantum* and *L. braziliensis* were not able to induce significant levels of those cytokines (Nogueira et al., 2020) but induced IL-10 (Castelli et al., 2019). Although this study was *in vitro*, *in vivo* studies with *L. amazonensis* EVs in B-1 cells and *L. infantum* confirmed their pro-inflammatory activity (Barbosa et al., 2018; Perez-Cabezas et al., 2019; Toledo et al., 2020). Pre-injection with EVs from those species induced cytokine production and increased parasite burden. Regarding *L. infantum*, proteomic analysis has suggested several molecules responsible for their functional activities (Santarem et al., 2013). However, a more detailed proteomic analysis was provided comparing procyclic and metacyclic-like forms (Forrest et al., 2020). Those analysis found approximately 50 virulence factors and some differentially expressed proteins in each stage. Most of the studies have focused on qualitative characterization of exoproteomes especially in *L. infantum* (Marshall et al., 2018). However, comparative exoproteome analysis from different *Leishmania* species are still scarce. For example, *L. infantum* and *L. mexicana* proteomics have been reported under the same conditions (Lynn et al., 2013). There is still a need for comparisons including a wider panel of *Leishmania* species. Those will provide information on how polymorphisms in the EVs protein could affect parasite-host interplay in different clinical forms. Collectively those findings strongly support the paramount role of exosomes released by various *Leishmania* spp. to trigger major immunomodulatory actions favorable for guaranteeing the infection and survival of the parasites in their hosts.

In a parallel study, exosomes released by macrophages infected with *L. mexicana* were found to be solely enriched in GP63 in terms of *Leishmania* proteins, whereas, as expected, the rest of the proteins corresponded to those of the macrophage (Hassani and Olivier, 2013). Macrophage exosomes released in response to LPS and *Leishmania* stimulation were found to induce macrophage inflammatory genes slightly differently in comparison to exosomes derived from naïve macrophages, reinforcing that EVs released during infectious context could be more prone to induce inflammatory reaction in the host.

#### Leishmania Exosomes and Sand Fly Midgut

One critical matter in the field of EVs, is to realize that the great majority of the studies performed in various fields (i.e. immunology, cancer, stem cells) were conducted with vesicles isolates from biological fluids or from cell cultures. However, Olivier’s team finally reported a pioneering finding showing that *Leishmania* EVs were being formed and freed within sand fly midgut and were delivered by *Leishmania* metacyclic promastigotes to the mammalian host during the blood feeding of the vector (Atayde et al., 2015). This co-transmission was revealed to be highly inflammatory in Balb/c mice and concurring to the development of hyper skin ulceration under a Th17-type response. Markedly, this study is the first to demonstrate *Leishmania* EVs enriched with GP63 are key sand fly-egested virulence factors, revealing *Leishmania* exosomes as critical infectious instruments for the proper development of leishmaniasis.

While *L. major* exosomes induce an IL-17a-mediated immune response (Atayde et al., 2015), Reiner and colleagues (Silverman et al., 2010a; Silverman et al., 2010b) found that IL-4 was a cardinal cytokine ruling over IL-17a in the exacerbation of skin inflammation. Of note, while in Reiner’s study, this phenomenon was observed in a context where exosomes were used for vaccination first prior to the challenge with promastigotes, Olivier’s lab experiments tried to mimic the physiological setup of the sand fly when taking a blood-meal and both exosomes and parasites are simultaneously transferred. Moreover, and according to previous reports, IL-17a is a very powerful signal to recruit neutrophil and to favor cutaneous leishmaniasis development in mouse and human skin pathology (Maurer et al., 2006; Boaventura et al., 2010; Griewank et al., 2014; Dietze-Schwonberg et al., 2019). Markedly, a study from Olivier’s laboratory strongly supports that *Leishmania* EVs concur to neutrophil migration toward the inoculation site (Hassani et al., 2014). Finally, several derived-insect components and parasite/bacteria were shown to be important during the sand fly bite (Dey et al., 2018). Those induced IL-1β induction *via* inflammasome by neutrophils and were important for *L. donovani* visceralization. Although a mixture of different components is found in the inoculum, the solely role of EVs in this process is an open field to be explored.

#### Hijack of Leishmania Exosomes by LRV1 Endovirus

Over the last 10 years, there have been several reports showing that certain *Leishmania* species belonging to the *Viannia* subgenus, which are lower eukaryotic organisms, can be infected by viruses. For instance. *L. (V.) guyanensis* is known to be infected by the endovirus *Leishmania* RNA Virus 1 (LRV1) (Guilbride et al., 1992; Stuart et al., 1992). Of note, this viral culprit within *Leishmania* was found to exacerbate the development of mucocutaneous leishmaniasis due to its capacity to trigger a strong TLR3/TLR7-dependent inflammatory response, which directly correlates with an enhancement in *Leishmania* metastatic behavior that is characteristic of mucocutaneous leishmaniasis (Ives et al., 2011). Consequently, understanding how this virus is transmitted is a critical step towards a better comprehension of not only *Leishmania* pathogenesis but also virus evolution and adaptation mechanisms.

Of interest, it is believed that LRV1 virus can propagate within *L*. (*V*.) *guyanensis* during their division, as well as to influence the host immune cells while dead parasites release the non-enveloped form of the virus (Olivier, 2011). While this remains a potential mechanism of viral propagation, some other pathways could be involved in such complex process. Olivier’s team recent observations using both *in vitro* cultures and *in vivo* sand fly vector approaches, showed that *Leishmania* promastigotes can actively produce and release exosomes in natural condition. Based on this discovery, they further investigated whether LRV1 could exploit *Leishmania* exosomal pathway to safely exit the promastigotes. Of utmost interest, they found that 30% of the exosomes released by *L*. (*V*.) *guyanensis* contained the whole LRV1 endovirus, conferring the virus protection against the different adverse conditions that it will encounter in the external milieu (Atayde et al., 2019a; Olivier and Fernandez-Prada, 2019). Additionally, LRV1 “exosomal shield” favored the virus to efficiently infect naïve *Leishmania* (*V*.) spp., which naturally fuse to and integrate EVs content to potentially gain information, as reported for *T. brucei* (Eliaz et al., 2017). However, in this case, naïve parasites rapidly become virally infected and see their level of infectivity modified, as previously discussed. In addition, LRV1 contained within *Leishmania* exosomes were found to be responsible for the TLR3-dependent induction of inflammasome and exacerbation of *L. (V.) guyanensis* infection (Barrias et al., 2019; Olivier and Zamboni, 2020).

Of note, although viral particles and exosomes are very alike, they share several physical and structural similitudes. A variety of viruses infecting humans have been found to use host-cell exosomes as vessels for transporting their nuclear materials and capsids, also impacting the overall viral pathogenesis and virus-induced subsequent pathologies (Meckes and Raab-Traub, 2011; Alenquer and Amorim, 2015). However, LRV1 using *Leishmania* exosomes is the first demonstration of exosomes harboring a whole virion. Overall, it is clear that *Leishmania* parasites of the *Viannia* subgenus and their LRV1 endovirus represent an impressive mutualistic relationship in which both entities are interconnected by exosomes.

#### Leishmania Exosomes in Drug Resistant Parasites

Control of leishmaniasis is based on a very short list of chemotherapeutic agents headed by pentavalent antimonials, followed by miltefosine and amphotericin B. These drugs are far from ideal due to host toxicity, elevated cost, limited access, and high rates of drug resistance (Leprohon et al., 2015; Olivier and Fernandez-Prada, 2019; Saha et al., 2020). As leishmaniasis is a vector-transmitted disease, the spread of drug-resistant parasites is ultimately dependent on their transmission potential for which within- and between-host ecology plays a key role. Markedly, as discussed through all this review, EVs play a major role in all these transmission/interaction events. Considering that the molecular content of eukaryotic EVs is a fingerprint of the origin cell reflecting its physiological/functional status, Fernandez-Prada’s laboratory recently explored the composition of leishmanial EVs in the context of drug resistance, in collaboration with Olivier’s group (Douanne et al., 2020). This was the first study describing *L. infantum* EVs’ core proteome, as well as all those proteins specifically enriched in EVs released by antimony-, miltefosine- and amphotericin-resistant parasites. Fernandez-Prada’s work showed for the first time that drug-resistance mechanisms can induce changes in the morphology, size, and distribution of EVs in *Leishmania*, with drug-resistant parasites releasing larger vesicles when compared to the wild-type counterpart (especially amphotericin B-resistant parasites with vesicles larger than 200 nm). Of note, several virulence factors (i.e. GP63), putative transcription factors (i.e. CBF/NF-Y), as well as proteins encoded by drug-resistance genes (i.e. antimony drug-resistance gene *mrpA* (Douanne et al., 2020) were identified among drug-specific enriched proteins. In relation to these last, MDR transporters were shown to transmitted from drug-resistant to drug-sensitive tumor cells by exosomes *in vivo* and *in vitro* (Lopes-Rodrigues et al., 2016), which points to a possible similar mechanism of drug-resistance transmission in *Leishmania* parasites. Currently ongoing and future studies will bring new knowledge on how EVs released by drug-resistant parasites contribute to drug resistance and, in a more general context, to the survival of *Leishmania* parasites through all the stressful conditions encountered during their life cycle.

## Future Applications and Therapeutic Use of Host and Trypanosomatid EVs

One major challenge for the near future is the development of new and improved therapies for the treatment and prevention of diseases caused by trypanosomes. The EVs from these parasites can offer new potential developments for diagnosis, follow-up of treatment responses, monitoring disease progression and determining the prognosis. EVs can also be helpful in devising new vaccine targets. For example, we have found that immunization mice with *T. cruzi* EVs obtained from trypomastigotes can be induce protection against experimental Chagas disease (unpublished). Nevertheless, the molecules found in EVs responsible for this protection are yet to be determined and will in turn, represent novel biomarkers. In addition, EVs isolated from patients can be a biomarker during early clinical stage, acute and chronic phase. These EVs can be associated with clinical stage or allow the follow up in clinical trials new drugs. Further investigation of these EVs regarding the micro-RNA and long coding RNA, for example, could provide relevant information about Chagas’s disease progression in humans. We have started to compare the circulating EVs isolated from plasma from patients with Chronic Chagas Disease (CCD). The plasma from CCD released less EVs with differences in their ability to induce cytokine production. CCD patients EVs were able to induce a differential production of IFN-γ and IL-17 in relation to controls, with differences being more evident in earlier/less severe stages of the disease (Madeira et al., 2020 submitted).

## Conclusions

**Figure 2:**
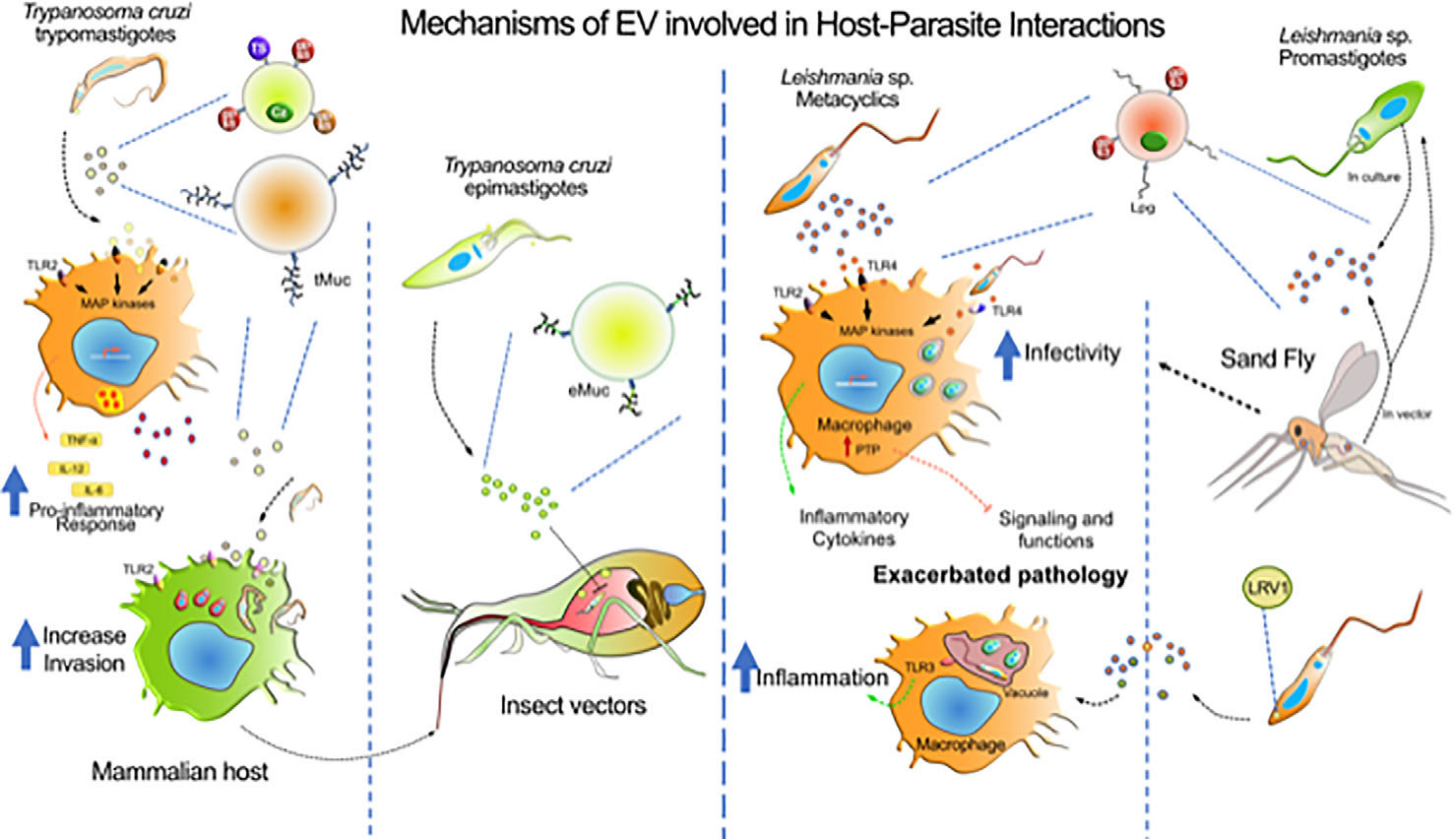
Mechanisms of EV involved in host-parasite interactions. The diagram represents the major contributions of EVs from *T. cruzi, Leishmania* sp. and host cells during the infection by these parasites. *T. cruzi* release heterogeneous populations of EVs containing surface mucin-like glycoproteins (tMuc and eMuc), GPI anchored proteins (GP63 protease, GP85, and TS superfamily members) and soluble proteins (cruzipain, Cz), which interact with macrophages through TLR2 receptors which activate MAP kinases and induce a pro-inflammatory response (TNF-α, IL-12, and IL-6). Macrophages also release EVs that might affect the host immune system. The TLR2—*T. cruzi* EVs interaction enhances further parasite invasion. In the insect vector, the parasite epimastigote EVs delays translocation of parasites through intestine. *Leishmania* metacyclics release EVs enriched with GP63 and LPG, that could interact with macrophages *via* TLRs and, depending on the species, to induce pro- and anti-inflammatory responses. GP63-enriched EVs were found to induce host protein tyrosine phosphatases concurring to alter macrophage signaling and microbicidal functions. However, receptors involved in EVs recognition concurring to augment infection of phagocytes is still not identify. *Leishmania* virus (LRV1) incorporate within Leishmania exosomes was found to induce myeloid cells (macrophage, neutrophil) inflammasome in a TLR-dependent fashion concurring to exacerbate skin pathology. *Leishmania* exosomes are released in the gut of the sand Fly vector and have been found to be co-inoculated with promastigotes during blood meal. Revealing that this co-inoculation can exacerbate myeloid cells infection and skin pathology development.

EVs studies increase every year, since they represent valuable biological markers (**Table 1**) with a powerful diagnostic and therapy function. Several works published in the literature show that the EVs released by trypanosomatids might play a fundamental role in the pathogenesis of CD, HAT and several *Leishmania* species and in the host’s immune response to the parasite (Trocoli Torrecilhas et al., 2009; Cestari et al., 2012; Torrecilhas et al., 2012; Santarem et al., 2013; Hassani et al., 2014; Atayde et al., 2015; Nogueira et al., 2015; Barbosa et al., 2018; Ribeiro et al., 2018; Toledo et al., 2020). These major effects are depicted in **Figure 2**. However, little is known about the mechanisms involved in the EV release process and whether this occurs as a result of damage to the parasite. In addition, the understanding of the relationship between different strains of *T. cruzi* (with different degrees of virulence) in the infectious process and in the production of EVs is still scarce. The same is valid for the different species of *Leishmania* that cause different type of diseases. Therefore, more studies are needed, to understand the EVs role in the pathogenesis of these parasitic endemic diseases, as well as in the mechanisms of pathogen-host interaction. This may be the starting point for the development of new preventive and therapeutic strategies with more efficient pharmacological targets against these parasitic which continues difficult to prevent, and treat and, whose current available drugs are toxic and not completely effective. The papers published in the literature, so far, show the important role that these vesicles play in the infectivity and in the modulation of the host’s immune response by the parasite, however the study in this field still has some gaps to be filled, mainly in functional biological aspects that allow a better understanding of the mechanisms and conditions for the release of these vesicles. Therefore, more studies are needed, as one of the fundamental points for the control of endemic parasitic diseases is the understanding of mechanisms involving the pathogen-host interaction.

## Author Contributions

All authors have been equally involved in writing and editing. Figures have been done by SS and AT, and edited in addition by MO. All authors contributed to the article and approved the submitted version.

## Funding

This work was supported by the SS and ACT FAPESP 2019/15909-0), ACT CNPq (408186/2018-6) and CAPES. Research in Olivier Lab is funded by grants from the Canadian Institute of Health Research (CIHR grant No. 159765) and the Natural Sciences and Engineering Research Council of Canada (NSERC). Research in Fernandez-Prada Lab is funded by a Natural Sciences and Engineering Research Council of Canada Discovery Grant RGPIN-2017-04480 and by the Canada foundation for Innovation (www.innovation.ca), grant number 37324.

## Conflict of Interest

The authors declare that the research was conducted in the absence of any commercial or financial relationships that could be construed as a potential conflict of interest.
